# An Added Strength

**Published:** 1900-11

**Authors:** 


### AN ADDED STRENGTH.

THE JOURNAL congratulates its contemporary, the *American Veterinary Review*, on the recent acquisition to its business management of Dr. Robert M. Ellis, of New York City. Dr. Ellis is among the most active veterinarians of the profession, with excellent qualifications to fill this important position, and we are sure the *Review* will have an added strength in every direction through the association of Dr. Ellis. We wish Dr. Ellis success and pleasure in his new field of work.

